# Meteorological Table

**Published:** 1813-03

**Authors:** 


### METEOROLOGICAL TABLE.

### *From January 25, to February 24, 1813.*

| D. | Therm. | | | Barom. | | Hygrom. *Dry.* | ——*Damp.* | | | Winds | Atmos. Variation. |
|---|---|---|---|---|---|---|---|---|---|---|---|
| 26 | 35 | 37 | 36 | $30^4$ | — | | 6 | — | — | NE.. | C... — ... —... |
| 27 | 36 | 38 | 35 | $30^4$ | — | | 6 | — | — | E..NE.. | C... — ... —... |
| 28 | 30 | 35 | 30 | $30^4$ | 3 | | 4 | — | 5 | NE.. | Fog... F.... —.... |
| 29 | 26 | — | 34 | 30[illegible] | $30^4$ | | 2 | — | 6 | NW... | C... |
| 30 | 33 | 36 | 35 | — | — | | 7 | — | 4 | NW | Fog.. |
| 31 | 38 | 40 | 39 | — | 5 | | 6 | — | 5 | NW | C.. |
| 1 | 37 | 39 | 38 | $30^4$ | 3 | | 5 | — | — | N | C.. —.. R... |
| 2 | 38 | 40 | 37 | — | — | | 6 | — | — | NE | Fog.. |
| 3 | 37 | 39 | 41 | — | 5 | | 7 | 6 | 5 | NW | F.. |
| 4 | 40 | 43 | 41 | $30^4$ | 3 | | 3 | 4 | 4 | N | C. |
| 5 | 40 | 42 | 39 | $30^3$ | $29^9$ | | 4 | 4 | 4 | NW | F. |
| 6 | 42 | 46 | 42 | $29^8$ | $29^9$ | | 6 | 5 | 4 | SE | F |
| 7 | 45 | 47 | 42 | — | — | | 4 | 7 | 8 | N | F. R.. F. R.. |
| 8 | 47 | 49 | 50 | $29^4$ | 6 | | 6 | 10 | 12 | W | R.. F. R. |
| 9 | 43 | 44 | 42 | — | 7 | | 6 | 3 | 7 | W | F.. R.. |
| 10 | 42 | 46 | 40 | 30 | — | | 6 | 4 | 5 | NE | F.. |
| 11 | 40 | 47 | 42 | — | $29^9$ | | 5 | 5 | 6 | N | F.. |
| 12 | 45 | 49 | 48 | $29^7$ | 4 | | 8 | 8 | 8 | NW | F.. |
| 13 | 45 | 50 | 42 | $29^5$ | — | | 6 | 5 | 8 | W | F.. R... F.. |
| 14 | 41 | 49 | 46 | $29^3$ | 2 | | 9 | 10 | — | S.. | R.. |
| 15 | 44 | 48 | 45 | 2 | — | | 9 | 12 | 9 | W... | R.. F. R... |
| 16 | 44 | 46 | 44 | $29^5$ | — | — 2 — | 4 | — | 4 | SW... | F... |
| 17 | 43 | 47 | 48 | $29^4$ | 2 | | 5 | 6 | 8 | S... | C.. |
| 18 | 44 | 49 | 44 | $29^5$ | 8 | — 5 — | 2 | — | 5 | W... | F.. R.. F.. |
| 19 | 44 | 50 | 52 | $29^5$ | — | | 8 | — | — | W.. | F. |
| 20 | 45 | 50 | 46 | $29^8$ | 9 | | 4 | 1 | 5 | SW... | F.. |
| 21 | 52 | 54 | 55 | $29^8$ | 7 | | 8 | 5 | 3 | W.. | C.. |
| 22 | 50 | 51 | 49 | — | — | | 1 | 2 | 4 | SW... | F.. |
| 23 | 44 | 47 | 41 | 30 | — | | 10 | 2 | 4 | NW.. | R... F.. |
| 24 | 42 | 46 | 41 | $30^1$ | 2 | | 4 | — | — | NW.. | F.. |

Quantity of rain from January 25 to February 24, two inches.

The prevailing winds, blowing in strong gales, in the latter part of the month, have been from the south and south-west. The thermometer has generally been high; but the mild temperature has not prevented an extraordinary occurrence of severe and obstinate catarrhal complaints: some instances of fatal peritoneal inflammations have happened in private practice. Rheumatism in the acute form is also rather prevalent.

